# Uncertainty analysis and performance evaluation of thermophysical property measurement of liquid Au in microgravity

**DOI:** 10.1038/s41526-023-00277-0

**Published:** 2023-05-24

**Authors:** Jannatun Nawer, Takehiko Ishikawa, Hirohisa Oda, Hideki Saruwatari, Chihiro Koyama, Xiao Xiao, Stephan Schneider, Matthias Kolbe, Douglas M. Matson

**Affiliations:** 1 Department of Mechanical Engineering, Tufts University, Medford, MA USA; 2 Institute of Space and Astronautical Science, JAXA, Tsukuba, Ibaraki Japan; 3 Human Spaceflight Technology, JAXA, Sengen, Tsukuba, Ibaraki Japan; 4 Institut für Materialphysik im Weltraum, Deutsches Zentrum für Luft- und Raumfahrt (DLR), Köln, Germany

**Keywords:** Characterization and analytical techniques, Mechanical properties

## Abstract

A new method for quantifying facility performance has been discussed in this study that encompasses uncertainties associated with thermophysical property measurement. Four key thermophysical properties: density, volumetric thermal expansion coefficient, surface tension, and viscosity of liquid Au have been measured in microgravity environment using two different levitation facilities. Levitation experiments were conducted using the Electrostatic Levitation Furnace (ELF) onboard the ISS in Argon and air, and the TEMPUS Electromagnetic Levitation (EML) facility on a Novespace Zero-G aircraft parabolic flight in Argon. The traditional Maximum Amplitude method was augmented through the use of Frequency Crossover method to identify the natural frequency for oscillations induced on a molten sample during Faraday forcing in ESL. The EML tests were conducted using a pulse excitation method where two techniques, one imaging and one non-imaging, were used to study surface oscillations. The results from both facilities are in excellent agreement with the published literature values. A detailed study of the accuracy and precision of the measured values has also been presented in this work to evaluate facility performance.

## Introduction

Facility performance evaluation is a continuous process of systematically evaluating the performance and effectiveness of one or more aspects of a system in relation to issues such as functionality, productivity, safety and security, accessibility, aesthetics, cost effectiveness, and sustainability. This study provides a systematic approach to evaluating facility performance through the use of uncertainty analysis. The measurement uncertainty in thermophysical properties has often been reported using a standard deviation or the half-width of an interval indicating the coverage probability which reflects the lack of complete information about the experimental conditions. Throughout the years, substantial effort has been directed towards development of reliable mathematical models which requires well characterized reference data for the properties of materials^1^. Uncertainty quantification can also help in understanding the variation in the quality of the manufactured parts using casting, welding and additive manufacturing^2^. So, it is crucial to report the associated uncertainties in the measured properties to accurately understand the error propagation in the predictive capability of mathematical models that use thermophysical properties.

A study of four key thermophysical properties: density, volumetric thermal expansion coefficient, surface tension, and viscosity was conducted utilizing two different levitation techniques: Electrostatic Levitation (ESL) and Electromagnetic Levitation (EML). The material in focus for this work is Gold (Au). Au is one of the pre-eminent noble metals which has been used for thousands of years to take advantage of its unique chemical and physical properties. The use of Au in a wide range of industrial applications has increased rapidly over the past decades and is now running at about the same level as the output both as pure metal and alloying components^3^. Its versatile and precise use in monetary, jewelry, electronics, medicine, and space applications have made it a perfect candidate for this evaluation. Au has been extensively studied over the years by various researchers and most of these studies do not contain a complete report on the associated uncertainties: Brillo^4^ reported a relative uncertainty of ~10% in the measured density and thermal expansion coefficient of liquid Au using EML; however, the author did not report uncertainties in mass, volume, and temperature; Paradis^5^ reported uncertainties in mass and volume during their density and thermal expansion coefficient measurements using ESL however they fail to report the uncertainties in temperature and variability in linear regression; Egry^6^ reported surface tension of liquid gold using EML, and Ofte^7^ reported viscosity of gold using oscillating cup viscometer with no report of associated uncertainties. In several recent publications, researchers have reported uncertainties with associated sensitivity for the measured properties using levitation techniques. Watanabe^8^ has reported a detailed uncertainty analysis of density of liquid Pt-X(X: Fe, Co, Ni, and Cu) alloys measured using EML; Jeon^9^ has reported a detailed uncertainty analysis of density of Ta, Mo and Nb using ESL; Moroshoshi^10^ has also reported uncertainty in surface tension measurement of liquid Fe. In this study, a detailed uncertainty analysis was conducted on each measured properties as a continuation of the facility comparison method previously developed^11^ by the author and is being extended to a different material class.

Processing of Au using levitation has its own challenges including the difficulty in achieving stable levitation of the sample in 1-g ESL. Terrestrial experiments are heavily influenced by strong 1-g force which induces deviations from the liquid sample’s spherical shape; most levitated sample analysis techniques are based on the assumption that the molten droplet is spherical. This material also has been reported to have higher work function than most other metals along with Pt and Pd^12^ which makes it difficult to remove electron from the solid surface during heating. EML testing induces significant stirring on ground and in a microgravity environment, and through MHD surrogate modeling it has demonstrated that gold will be turbulent under most operating conditions^13,14^. ESL in space has the advantage of negligible induced convection compared to EML. Thus, by studying this material in both ESL and EML, it is also possible to gain insight on the accuracy and precision of measured properties over a wide range of convection conditions.

## Results and discussion

### Density and volumetric thermal expansion coefficient

Density (*ρ*) and volumetric thermal expansion coefficient (*β*) of Au were determined by optical measurements using high-speed video recording in ISS-ELF. This process involved simultaneously recording video images and the temperature (*T*) of the sample during free cooling. The recorded video data from ISS-ELF were downloaded to earth for post-processing in a compressed MPEG-TS format which was also interlaced at 30 fields per second for increasing the apparent frame rate without consuming extra bandwidth. A custom computer algorithm was used to de-interlace the frames using spatial line doubling technique. After de-interlacing, no significant changes («1%) in the sample volume was observed. Sample background was also noticed to be brightened by ~0.6% which also influenced the edge detection. Dynamic mass (*m*) was tracked throughout the cycle for any mass loss or gain during the testing. Sample volume (*V*) was measured from the detected sample radius (*r*) using sub-pixel edge detection for higher accuracy.

1 $$\rho = \frac{m}{V}$$

2 $$\beta = \frac{1}{V}\left(\frac{{\partial V}}{{\partial T}}\right)$$

**Fig. 1:**
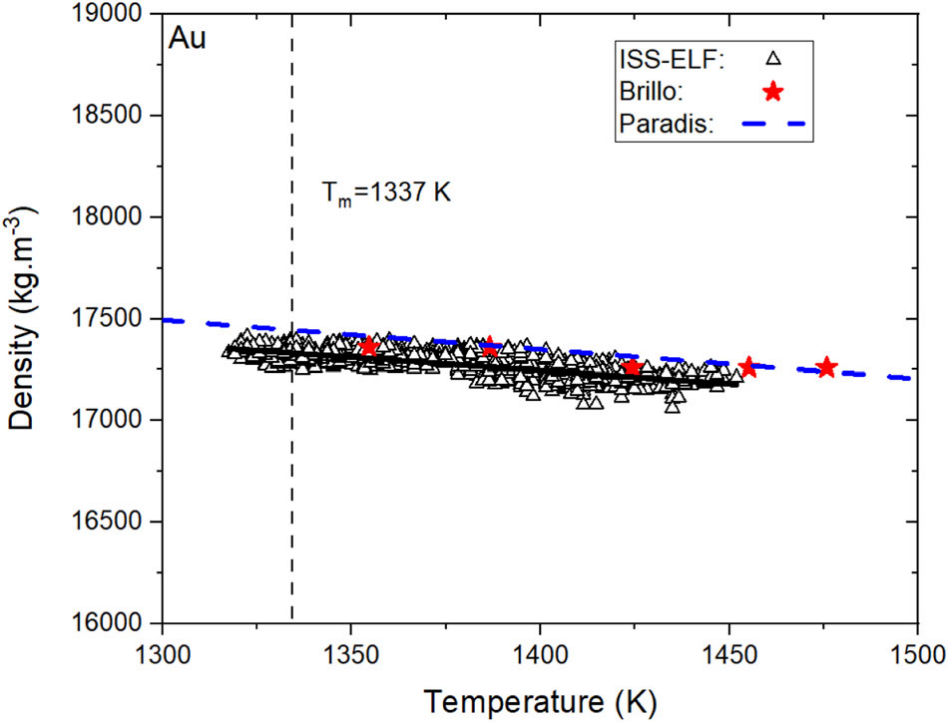
Density of liquid Au processed in ISS-ELF facility as a function of temperature. The solid line represents the linear fit obtained from the data.

The density of liquid Au was measured using the ISS-ELF in air environment and the results are shown in Fig. 1. The measured density showed a linear behavior as a function of temperature from the range of (1320–1450) K as:

3 $$\rho \left( T \right) = \left( {17327.64 \pm 1.66} \right) - (1.30 \pm 0.02)(T - 1337) {{{\mathrm{kg}}}}\cdot{{{\mathrm{m}}}}^{ - {{{\mathrm{3}}}}}$$

The measured density showed an excellent agreement with the published values as reported by Paradis^5^ and Brillo^15^ with less than 1% of deviation at the melting point. The slope of the linear regression of ISS-ELF density are also within 17% of the published values^4,10^. The measured volumetric thermal expansion coefficient from this study is (7.6 ± 1.7) ×10^−5^ K^−1^. The variability of the measured volumetric thermal expansion coefficient value encompasses the value reported by Zhang et al.^16^.

### Complementary natural frequency identification methods: max. amplitude and frequency crossover

**Fig. 2:**
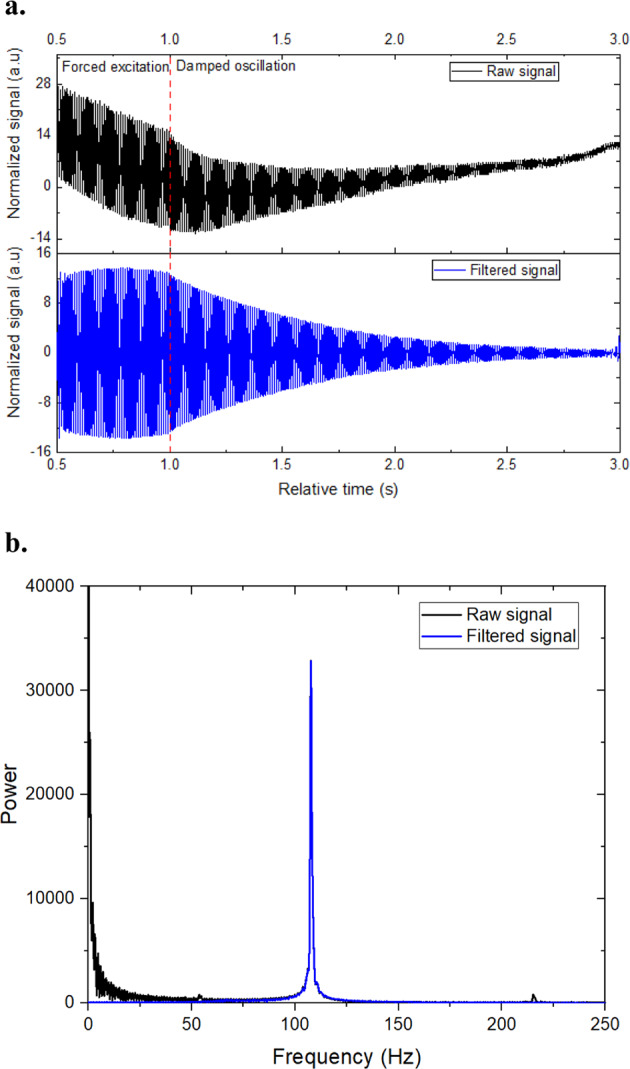
Droplet oscillation data analysis. **a** A typical signal (raw and filtered) of an Au sample processed in air at 3.0 kV excitation voltage in ISS-ELF, **b** FFT results of the analysis.

Surface tension and viscosity of Au in the liquid phase were measured on the ISS-ELF facility using oscillating drop technique. This facility employs a non-imaging method for detecting sample response to the excitation force through a circular photo detector which detects the change in extent of the shadow of the droplet created by a collimated beam^17^. Faraday forcing^18^ was employed to investigate the natural resonant frequency where a frequency sweep was conducted incrementally around the expected natural frequency with a step size of 2 Hz. A bandpass filter was applied to the raw signal to remove any low-frequency and high-frequency translational tones. Figure 2a shows forced, and free damped oscillation of a typical raw and filtered signal obtained during a Au experiment using the ISS-ELF. Forced signal corresponds to the sample oscillation which is observed under the forced resonant oscillations when the excitation voltage was turned on and damped frequency corresponds to the free oscillation which is observed after the excitation voltage is turned off. Analyzing deformation during forced oscillation provides a method to identify the maximum observed amplitude during forced excitation near the natural frequency. When excitation is turned off, oscillation shifts are observed, and evaluation of damping behavior provides an insight on the decay constant. Figure 2b shows the Fast Fourier Transformation (FFT) results of the free damping of the signal.

**Fig. 3:**
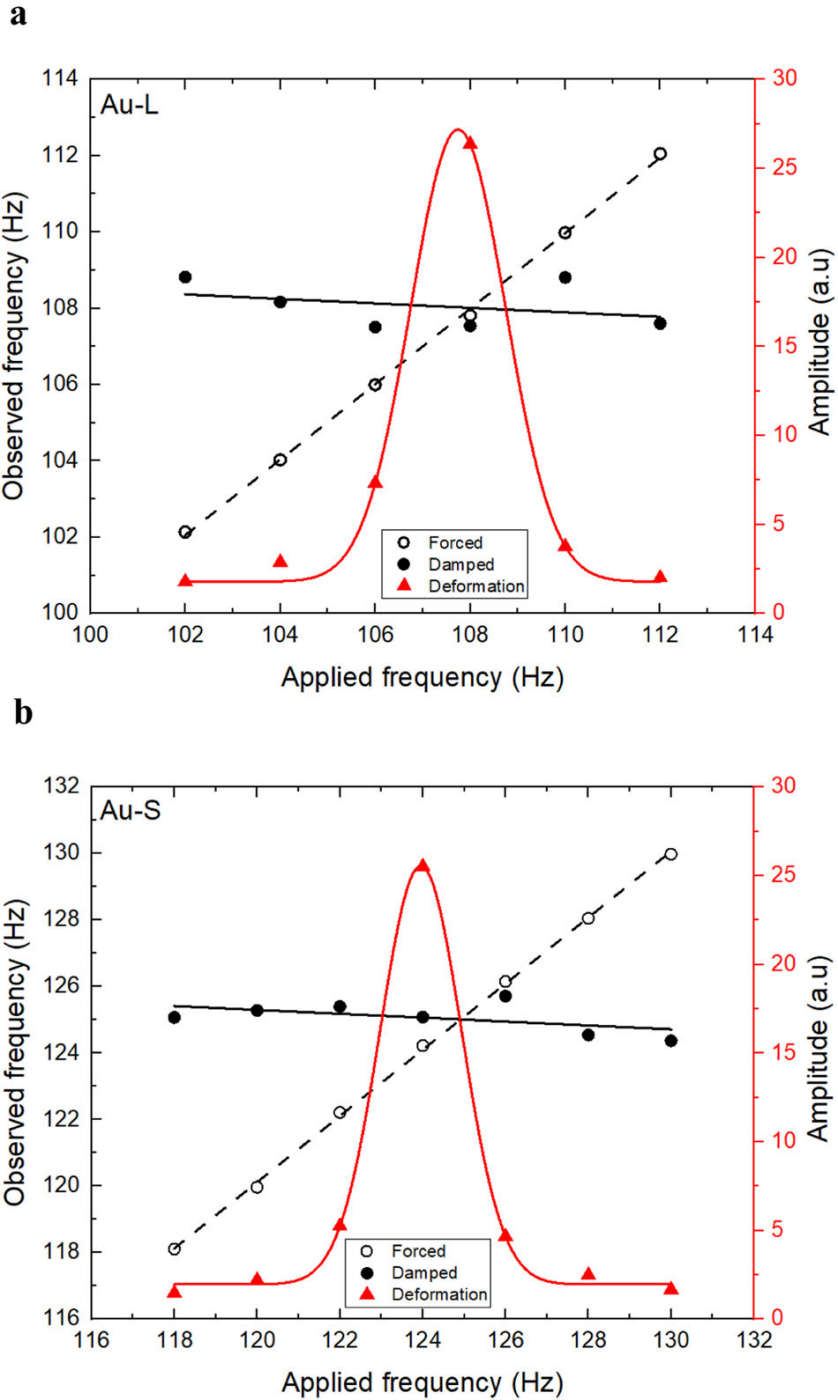
Frequency sweep conducted on the ISS-ELF. **a** Frequency sweep on a 80.7518 mg sample at 1525.77 K. **b** Frequency sweep on a 59.5434 mg sample at 1412.07 K. Forced frequency is represented using open circles, damped frequency is represented using closed circles and peak-to-peak amplitude is represented using red triangles.

Natural frequency (*f*_*n*_) was identified during post-processing using the Frequency Crossover (FC) method^6^ in parallel with the traditional Max. Amplitude (MA) method^11^. Figure 3 depicts the quantitative representation for both methods during a frequency sweep conducted on two different sized Au samples processed using the ISS-ELF. An increase in the projected amplitude of the forced excitation signal was observed with an increase in the forcing frequency. Simultaneously, a decrease in the damped frequency was observed as the resonant frequency was increased. Sample natural frequency was identified at the intersection of the linear trends of the forced and damped frequencies. For the larger sample as shown in Fig. 3a, the (FC) crossover was observed at 108.02 Hz. This finding was confirmed by the observed maximum projected amplitude at 107.72 Hz. For the smaller sized sample as shown in Fig. 3b, the FC value was 124.90 Hz, and the MA value was 123.96 Hz. Once the natural frequency was identified and the decay constant (*τ*) from the mode (2,0) oscillations were calculated, these values were used to evaluate surface tension (*σ*) and viscosity (*η*):

4 $$\sigma = \frac{{3\pi mf_n^2}}{8}$$

5 $$\eta = \frac{{\rho r^2}}{{5\tau }}$$

The linear regressions on surface tension and viscosity datasets can be expressed as:

6 $$\sigma = \left( {1.110 \pm 0.004} \right) - \left( {8.864 \pm 2.287} \right) \times 10^{ - 5}\left( {T - 1337} \right){{{\mathrm{N}}}}\cdot{{{\mathrm{m}}}}^{ - 1}$$

7 $$\eta = \left( {4.413 \pm 0.549} \right)\exp \left( { - \frac{{2.588 \pm 6.842}}{{T}}} \right){{{\mathrm{mPa}}}}\cdot{{{\mathrm{s}}}}$$

Surface tension was measured based on the evaluation of the natural frequency from both of these methods. A F-test was conducted to compare the slopes and intercepts of both FC and MA and are shown in Table 1. The *P*-value for the slope is 0.648 and for the intercept is 0.240. Since, both *P*-values are greater than the significant level of 5%, these slope and intercept values are not statistically different.

**Table 1:** F-test conducted to compare slope and intercept.

Parameters	F-value	Numerator DF	Denominator DF	*P*-value
Slope	0.16917	1	24	0.68450
Intercept	1.44417	1	25	0.24073

**Fig. 4:**
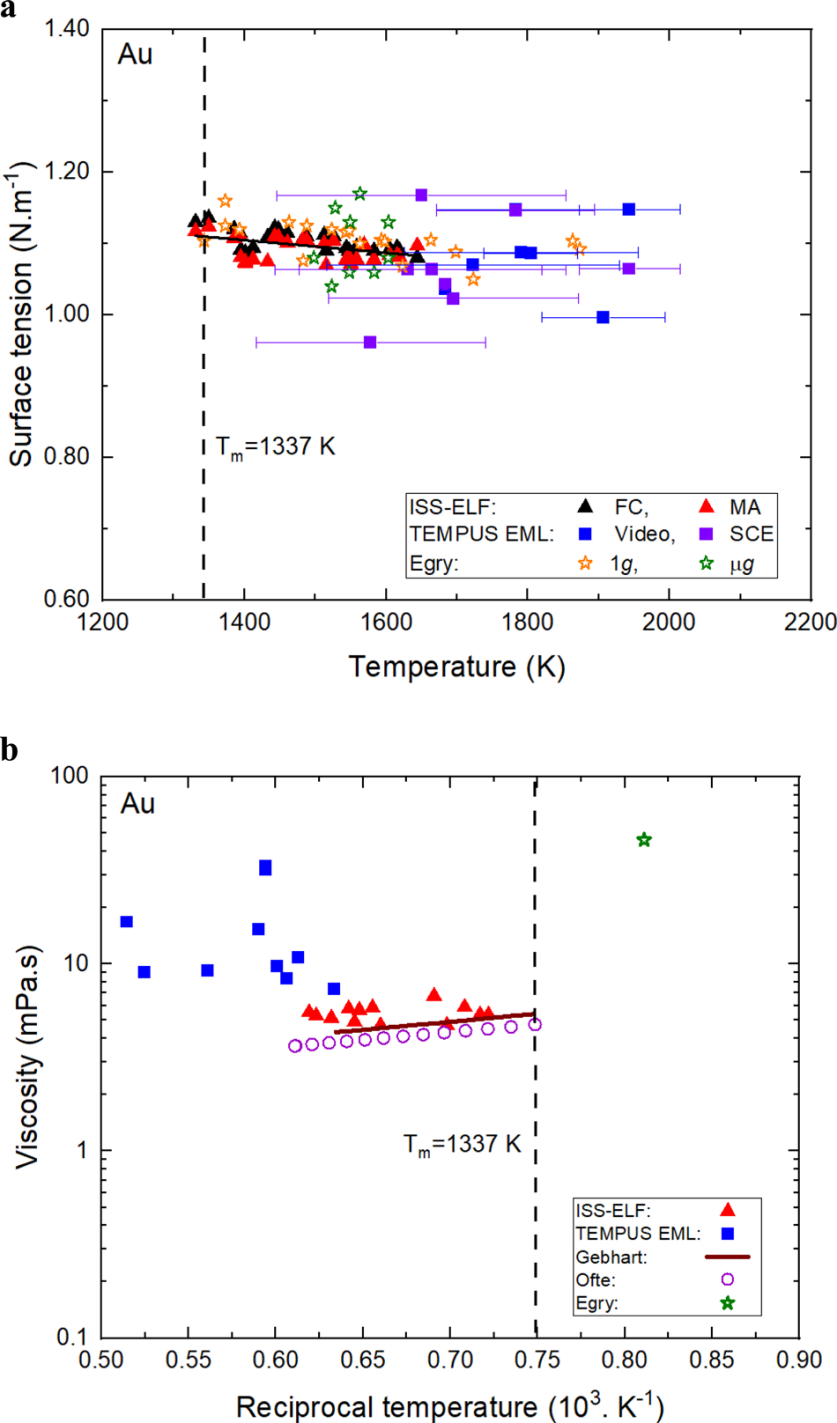
Droplet oscillation results of liquid Au. **a** Surface tension as a function of temperature. Error bars in the temperature are shown in terms of standard deviation**. b** Viscosity as a function of reciprocal temperature.

Surface tension values measured from both methods were used for an overall linear regression as shown in Eq. (6) and are shown in Fig. 4a as a function of temperature. The measured values from ISS-ELF show good agreement with Egry’s^6^ reported values from the IML-2 Spacelab mission TEMPUS EML facility. To validate this finding, these results are compared with the findings from pulse excitation methods conducted using the TEMPUS EML facility processed during parabolic flight testing. A 2.1862 g Au sample was processed during eight successful parabolas. Data from the parabolic flight has been analyzed using both video and Science Coupling Electronics (SCE)^19^ data and the difference is within 6% of each other. The measured values are highly scattered possibly due to significant sample translation which imposes off-mode excitation of the sample surface. The temperature variability is also large, which detrimentally enhances the error bars. Hence the surface tension behavior measured from this facility is inconclusive.

Viscosity values measured from this study has been fitted to Arrhenius Eq. (7) as a function of temperature. Figure 4b shows a semi-log plot of viscosity of liquid Au vs reciprocal temperature compared with published literature values. In space, where external forces are minimized as compared to conditions on earth, mode (2,0) oscillations were used to measure viscosity. The Au samples processed during parabolic flight did not completely dampen out during the short microgravity window and frequency beats were observed during damped oscillation. However, during the ISS-ELF testing with continuous microgravity environment this issue was not detected, and clean oscillations were observed. The measured values from ISS-ELF are in excellent agreement with Ofte’s^7^ reported viscosity values using oscillating cup viscometer and Gebhart’s^20^ reported viscosity values using the rotating cylinder method. The measured viscosity values from the parabolic flight are a magnitude higher than the values measured in the ISS-ELF. However, they are in the same order of magnitude range as Egry’s^21^ single reported data point from IML-2 space testing. As observed with higher reported value of viscosity by Egry^21^, the sample was always turbulent, even during cooling as shown by Baker^13^. The impact of turbulence also increases viscosity in a systematic manner as shown by Xiao^22^.

## Uncertainty analysis

The evaluation of uncertainty for this study has been conducted according to the Evaluation of Measurement Data-Guide to the Expression of Uncertainty in Measurement (GUM 1995) principal handbook^23^. For each of the properties measured, the variables associated with the measurement have been considered during uncertainty analysis including the variability of the coefficients of the linear regression models. In this study, a sample calculation on uncertainty of the properties at the melt (1337 K) are shown Table 2 and the details are discussed in the following subsections.

**Table 2:** Relative standard uncertainty (*u*) and combined standard uncertainty (*u*_*c*_) of Au at the melting point (T_m_ = 1337 K).

Factors (%)	ISS-ELF	TEMPUS EML
Uncertainty of temperature: *u(T)/T*	1.59	0.42
Uncertainty of mass: *u(m)/m*	0.43	0.01
Uncertainty of radius: *u(r)/r*	0.56	3.68
Uncertainty of volume: *u(V)/V*	1.70	11.04
Uncertainty of slope: $u{\left({\frac{{\partial V}}{{\partial T}}}\right)}\big/{\frac{{\partial V}}{{\partial T}}}$	2.30	—
Uncertainty of frequency: *u(f*_*n*_*)/ f*_*n*_	0.57	1.95
Uncertainty of time constant: *u(τ)/ τ*	10.81	31.46
Combined uncertainty of density: *u*_*c*_(*ρ*)/*ρ*	1.75	—
Combined uncertainty of volumetric thermal expansion coefficient: *u*_*c*_(*β*)/*β*	2.86	—
Combined uncertainty of surface tension: *u*_*c*_(*σ*)/*σ*	1.22	3.91
Combined uncertainty of viscosity: *u*_*c*_(*η*)/*η*	10.39	32.31

### Uncertainty in density and volumetric thermal expansion coefficient measurement

The uncertainty of the density measurement was evaluated by considering the uncertainties in both mass and volume measurements:

8 $$\frac{{u_c(\rho )}}{\rho } = \sqrt {\left(\frac{{u(m)}}{m}\right)^2 + \left(\frac{{u(V)}}{V}\right)^2}$$

9 $$\frac{{u_c(\beta )}}{\beta } = \sqrt {\left(\frac{{u(V)}}{V}\right)^2 + \left(\frac{{u(\frac{{\partial V}}{{\partial T}})}}{{\frac{{\partial V}}{{\partial T}}}}\right)^2}$$

Uncertainty in sample mass was calculated from the accuracy of the laboratory mass balance and uncertainty in sample evaporation. For an ESL sample of 80.75 mg processed in air using ISS-ELF, the relative uncertainty in mass is 0.43% and for an EML sample of 2.1862 g processed in Argon at TEMPUS EML, the relative uncertainty is 0.01%. The volume of the spherical sample was obtained from a calibration factor which is the ratio of the volume of calibration sphere to that of the pixel count of the calibration from the image analysis determined though edge detection. The combined standard uncertainty in the volume measurement has been calculated using the uncertainty obtained from the accuracy of calibration spheres and from the uncertainty in the volume associated with edge detection. The relative combined uncertainty in volume for ISS-ELF sample is 1.70% and for TEMPUS EML is 3.58%. The uncertainty in $\frac{{\partial V}}{{\partial T}}$ from ISS-ELF is 2.30% which has been evaluated from the variability of the slope of the linear fit while considering the appropriate propagation of error during the calculation.

Employing the uncertainty in measurement techniques, ELF experiments at the melting point indicate a liquid density of 17,400 ± 304 kg.m^–3^ and a volumetric thermal expansion coefficient of 7.60 ± 0.217 × 10^–6^ K^–1^. Density and thermal expansion coefficient were not measured in TEMPUS EML during this study.

### Uncertainty in surface tension measurements

Several factors can affect surface tension measurement during levitation experiments such as material purity, oxygen solubility in the samples, gasification, evaporation, and surface oxidation or nitriding from residual gases. Melt contamination is possible and can dramatically impact the surface tension. Uncertainty of the measured surface tension can be evaluated from the relevant uncertainties in mass and frequency:

10 $$\frac{{u_c(\sigma )}}{\sigma } = \sqrt {\left(\frac{{u(m)}}{m}\right)^2 + 4\left(\frac{{u(f_n)}}{{f_n}}\right)^2}$$

The uncertainty in the frequency can mainly arise due to the resolution of frequency and from sample rotation^24^. A determination of sample rotation from the video data of ISS-ELF was not possible to quantify due to the limitations of optical equipment during sample oscillation. So, the uncertainty of sample deformation is assumed to be negligible for this study. The relative uncertainties in frequency at the melting point for ISS-ELF sample is 0.57% and the TEMPUS EML is 1.95%. So, the combined uncertainty in surface tension value of 1.11 N.m^−1^ measured in ISS-ELF is calculated to be 0.01 N.m^−1^ and the combined uncertainty in surface tension value of 1.01 N.m^−1^ measured in TEMPUS EML is calculated to be 0.03 N.m^−1^.

### Uncertainty in viscosity measurements

Viscosity is a function of density, sample radius and time constant and according to GUM, the global uncertainties in the measurement can be determined from:

11 $$\frac{{u_c(\eta )}}{\eta } = \sqrt {\left(\frac{{u(\rho )}}{\rho }\right)^2 + 4\left(\frac{{u(r)}}{r}\right)^2 + \left(\frac{{u(\tau )}}{\tau }\right)^2}$$

The experimental uncertainty in the time constant from sample rotation was assumed to be negligible so only the error from the linear fit^25^ was used to measure uncertainty in time constant. In the aperiodic relaxation, all the recorded datapoints used in the fitting procedure have been used to find the relative standard error for each small time-segment and the average of all the errors have been used to quantify the uncertainties in the time constant. Since density was not reported in the TEMPUS EML, a literature density value was used for the viscosity analysis assuming no variability present in the data. The relative standard uncertainty of time constant in the ISS-ELF is calculated to be 10.18% and in TEMPUS EML it is 31.46%. So, the combined uncertainty in measured viscosity of 5.6 mPa.s in ISS-ELF is 0.58 mPa.s and the combined uncertainty in measured viscosity of 10.65 mPa.s in TEMPUS EML is 3.44 mPa.s. Since the uncertainty in density and radius are relatively smaller, the dominant contributing factor to this combined uncertainty is from the uncertainty in decay constant. Decay constants were evaluated differently for the two facilities. For the TEMPUS EML, an overall exponential fit was utilized based on the entire decay envelope which contributed to higher uncertainty in the time constant measurement. In contrast for ISS-ELF, the decay envelope was segmented into smaller discrete time intervals from which the evolution of the decay signal could be tracked^26^. This discretization resulted in a smaller uncertainty for time constant measurement. This is an important distinction to raise as error investigation involves quantification not only of the precision of the measurement technique, but also the error from selection of a specific analysis protocol.

### Uncertainty in temperature measurements

Some researchers have reported a fixed temperature uncertainty over the entire temperature range of (5–10) K^27^ which is a reasonable assumption in cases involving small superheating or undercooling as indicated by noise free temperature readings. However, this does not address the uncertainty associated with some sample specific experimental temperature measurement for each facility used.

**Fig. 5:**
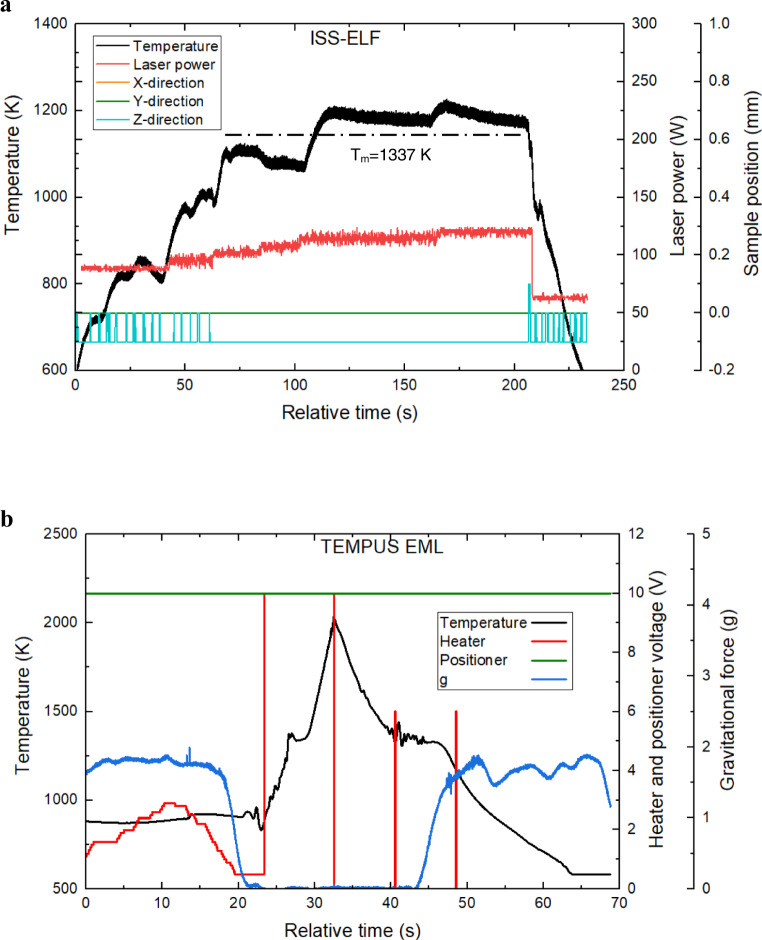
Typical thermal cycles of Au processed in two microgravity facilities. **a** Thermal profile of a sample processed using ISS-ELF along with laser power and sample position. **b** Thermal profile of a sample processed using TEMPUS EML along with heater and positioner settings, and the gravitational force.

In ISS-ELF, the melt and recalescence were difficult to identify due to the sample motion induced noise in the recorded temperature as evident from Fig. 5a which shows a typical thermal profile of Au from the ISS-ELF testing along with the total laser power and sample position in X, Y, and Z direction. Melt plateau was identified by analyzing both pyrometer and video data for this facility. In TEMPUS EML, sample movement was restricted by strong electromagnetic force and the recorded temperature shows less noise as shown in Fig. 5b. The uncertainty in the temperature measurement can be calculated using:

12 $$u_c(T) = T^2\sqrt {\left(\frac{{u(T_P)}}{{T_P^2}}\right)^2 + \left( - \frac{{u(T_{PL})}}{{T_{PL}^2}}\right)^2}$$

where, *T*_*P*_ corresponds to the raw temperature and *T*_*PL*_ corresponds to the observed liquidus temperature. The uncertainty in observed liquidus temperature in ISS-ELF is 15.05 K and in TEMPUS EML is 4.47 K. The combined uncertainties in melting point temperature in ISS-ELF is 21.32 K and in TEMPUS EML is 5.61 K.

### Facility comparison

Comparative measurement based on high accuracy and precision evaluation involving different measurement methods are important in understanding how a facility performs for a given material classification. This could also serve as the basis for prioritization of specific ground and space thermophysical property investigations. From the detailed study of the uncertainties measured during this work, accuracy, and precision of each measurement have been calculated. Accuracy has been measured in the form of deviation from a literature value which is highly dependent on the literature value selected for the comparison. For this study, previously reported thermophysical property values measured using levitation techniques have been utilized for consistency. Precision has been measured by considering combined uncertainties mentioned in the previous sections and the variability in the linear regression of the final reported datasets where *μ* corresponds to the average value of the dataset,

13 $$Deviation = \frac{{\mu _{measured} - \mu _{literature}}}{{\mu _{literature}}}$$

14 $$Coefficient\;of\;variation = \frac{{\sqrt {u_c^2 + u_{regression}^2} }}{{\mu _{measured}}}$$

**Fig. 6:**
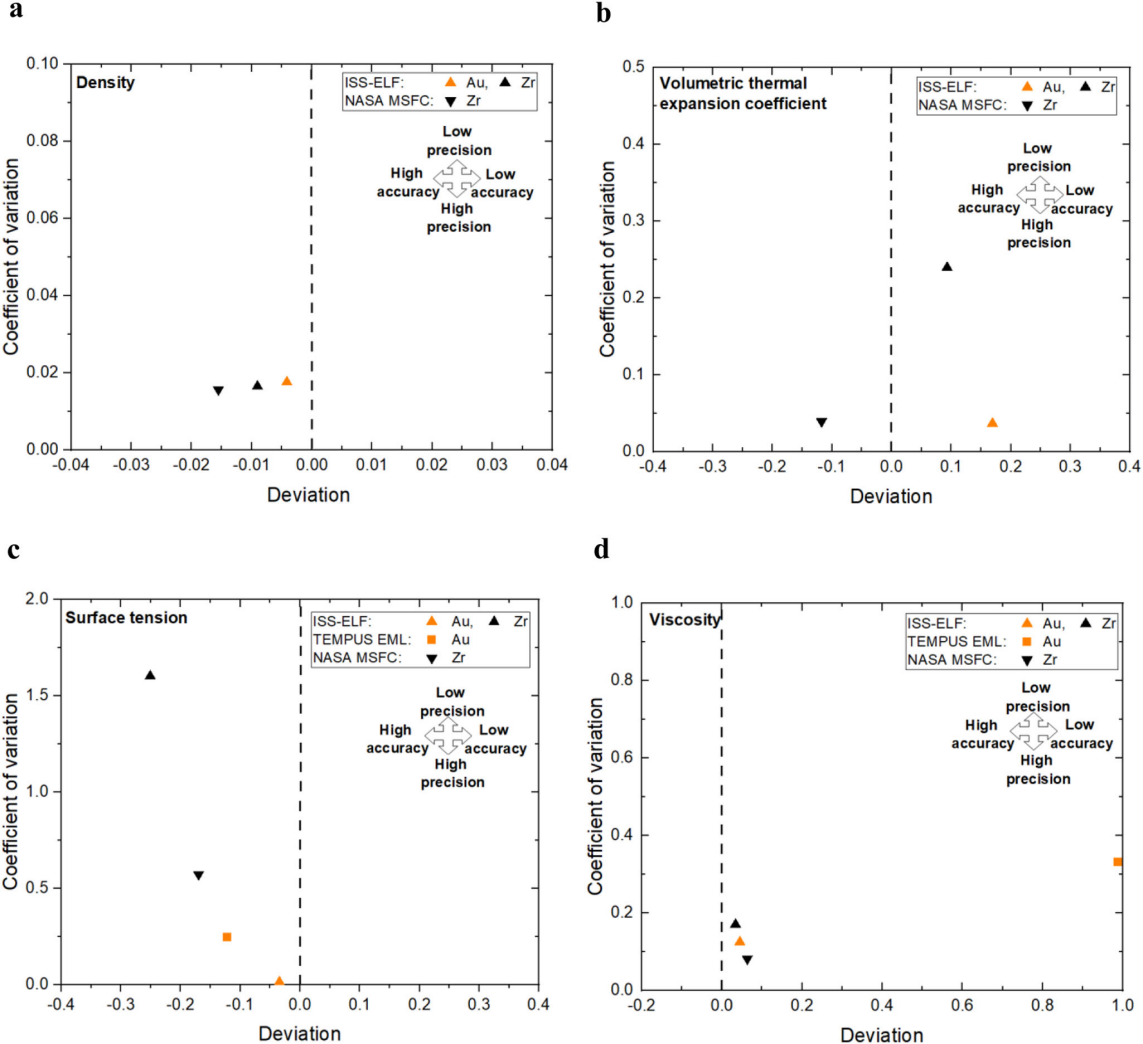
Accuracy vs precision of the measured properties at the melting point of Au (1337 K). Measurement accuracy and precision of **a** density, **b** volumetric thermal expansion coefficient, **c** surface tension, and **d** viscosity of liquid Au are compared with Zr processed in ISS-ELF and NASA MSFC ESL facilities. The triangles represent results from space-based ISS-ELF, the inverted triangles represent ground-based NASA MSFC ESL results, and the rectangular markers represent the results from the parabolic flight TEMPUS EML.

Accuracy and precision measurements for all four properties of Au at the melting point from this study are shown in Fig. 6 along with previously reported Zr values^11^. In Fig. 6a, b, deviation from literature value of density and thermal expansion coefficient has been calculated with respect to previously reported density value of 17.40 × 10^4^ kg.m^−3^ and thermal expansion coefficient value of 7.60 × 10^−5^ K^−1^ by Paradis^5^. The Au density measured in ISS-ELF has similar accuracy and precision as the Zr density measured in ISS-ELF and NASA MSFC ESL^11^. The liquid volumetric thermal expansion coefficient of Au measured in ISS-ELF has similar precision as the Zr values measured in terrestrial facility. This confirms that the findings from this study are comparable to ground-based studies.

In Fig. 6c, d, deviation of surface tension and viscosity has been compared with previously reported (baseline reference) surface tension value of 1.15 N.m^−1^ and viscosity value of 5.36 mPa.s by Gebhart^20^. Surface tension and viscosity values of Au measured in the ISS-ELF have much higher accuracy and precision as compared to the values measured in TEMPUS EML. The extended microgravity condition and controlled frequency sweeps during Faraday forcing in ISS- ELF provided the opportunity to measure high quality surface tension and viscosity data in this facility. Thus, it is appropriate to focus on the precision values from these plots as these values are entirely measured from the specific experiments run as a part of the current study.

Temperature is the major contributing factor to the uncertainties of all the measured properties in this work. ISS-ELF has the higher overall uncertainties in temperature due to sample motion which caused the sample to be in and out of pyrometer field-of-view due to reduced electrostatic force required during the ESL testing in space. Larger sample size and strong positioning forces in TEMPUS EML reduces the impact from sample motion. Hence, the sample appears more stable in this facility with less noise as compared to ISS-ELF. However, short window of microgravity during the parabolic flight are not optimum for the sample to damp out properly and ISS-ELF is best suited for processing viscous samples such as Au. The relevance of any performance evaluation will obviously be governed by the reliability of the experimental data on which it is based on and through this study an attempt has been made to provide a meticulous evaluation of the uncertainty of the measurements. The ISS-ELF facility was initially designed to process high temperature refractory materials^17^. This study has shown that this facility can not only process metallic samples over a wide temperature range but also can produce high quality thermophysical property data. The TEMPUS EML facility exhibited excellent temperature control and sample stability due to larger sample size and higher positioning force. The non-image-based SCE data for oscillation analysis could be used alongside the video data acquisition method; this topic will be further explored in a future paper.

Density, volumetric thermal expansion coefficient, surface tension, and viscosity of liquid Au have been successfully measured under microgravity condition during this study. The reported values measured on the ISS-ELF during this study showed excellent agreement with reported literature values. Viscosity measured in TEMPUS EML are an order of magnitude higher than the reported viscosity values from ISS-ELF and literature values. Unlike accuracy, which requires a vast knowledge and understanding of the quality of the literature values, this study concentrates on an evaluation of precision which was measured from the uncertainty analysis. The new methods presented here can be used to successfully evaluate facility performance as shown in Fig. 6. In conclusion, a new quantitative graphical comparison technique has been developed and utilized for facility comparison highlighting accuracy and precision of Au. The high accuracy and precision properties measured in the ISS-ELF shows that this facility is one of the best available tools for processing this material using levitation methods.

## Methods

Au has been studied in two ESL and EML facilities. The raw material for this study were obtained from Sure Pure Chemetals Inc. with a purity of 99.99%. The ESL samples for this study were prepared to accommodate facility requirements (1.8–2.0 mm). EML samples studied in this work is much larger with a sample size of around 2 g.

### Experimental facilities

**Fig. 7:**
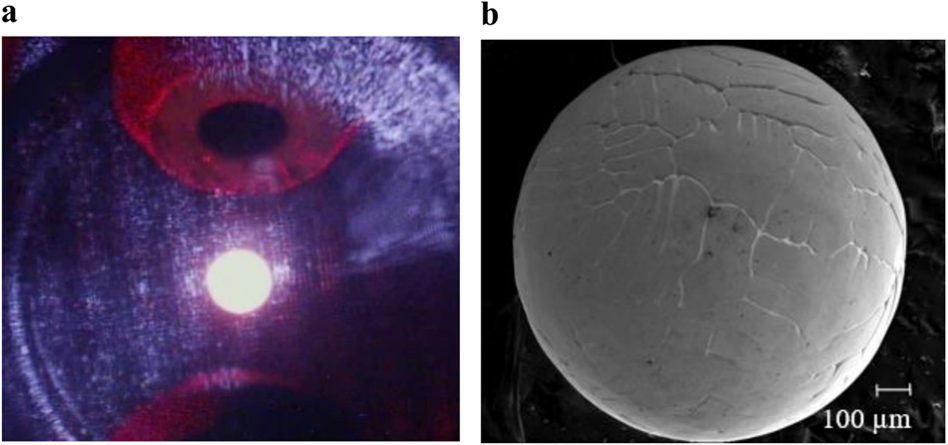
Images of Au sample. **a** A levitated Au sample in the ISS-ELF facility. **b** An SEM image of a processed Au sample from the ISS-ELF.

The space testing was conducted using the Japan Aerospace and Exploration Agency’s (JAXA) Electrostatic Levitation Furnace (ELF) onboard the International Space Station which is known as the ISS-ELF. Detailed description of this facility has been reported by Tamaru et al.^28^. A typical Au sample levitated at this facility is shown in Fig. 7a. This facility utilizes image-based methods for density/thermal measurements and a non-imaging area array method for tracking droplet oscillation and damping expansion circular behavior. The power meter in this facility detects sample deformation through the fluctuation of laser power and has a sampling rate of 5000 Hz with a total of 5 s signal recording time. Processed Au in air allowed a maximum excitation voltage application of 3 kV. Operations in this facility were conducted remotely from the ground using telescience; after each test, the data was downloaded for further analysis. All surface tension analyses using both FC and MA methods are reported as part of this work. Based on the observations from the frequency sweeps as shown in Fig. 3, viscosity values are being reported for a threshold of an amplitude of 19 a.u or greater. Any frequency sweep which exhibited maximum amplitude below this threshold was not fully excited to mode 2,0 and often exhibited mixed mode oscillations. Post-processing surface analysis was conducted using Scanning Electron Microscopy (SEM) at the Institute of Materials Physics in Space DLR, Cologne as shown in Fig. 7b.

**Fig. 8:**
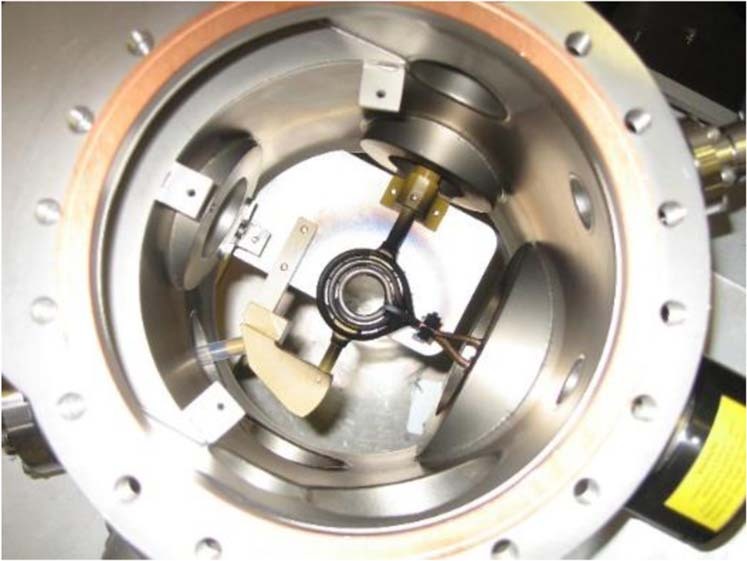
TEMPUS EML facility. Parabolic flight test chamber onboard the Novespace-Airbus A-300 “Zero-G” aircraft.

EML experiments were performed on the TEMPUS (Tiegelfreies Elektromagnetisches Prozessieren von Proben unter Schwerelosigkeit) facility operated by the German Space Center Deutsches Zentrum für Luft- und Raumfahrt (DLR) as shown in Fig. 8. This levitation facility provides a short window of microgravity (~23 s) during parabolic flights onboard the ESA A310 aircraft^29^. A typical experiment consisted of a single melt cycle during each parabola as shown in Fig. 5b. The sample was preheated to just below the melting point during the 2 g ascent phase of the parabola. When microgravity was achieved, the sample was fully molten and then allowed to cool. Excitation is conducted during the free fall phase of each parabola. The Au samples were processed in 350 mbar of Argon gas atmosphere. For this study, an inductive measurement hardware device called the Sample Coupling Electronics (SCE) with a data acquisition rate of 400 Hz has been utilized^30^ to augment visual observations. Traditionally, this device is used for measuring electrical resistivity of a sample; however, surface tension and viscosity can also be measured from the resonant circuit frequency response.

## Data Availability

The data that support the findings of this study are available from the corresponding author upon request.

## Code availability

The custom image analysis algorithms used during the course of this study are available from the corresponding author upon request.

## References

[1] Bambach M, Fugenschuh A, Buhl J, Jensch F, Schmidt J (2020). Mathematical modelling and optimization for powder-based additive manufacturing. Proc. Manuf..

[2] Hu Z, Mahadevan S (2017). Uncertainty quantification and management in additive manufacturing: current status, needs, and opportunities. Int. J. Adv. Manuf. Technol..

[3] Corti, C. & Holliday, R. *Gold: Science and Applications* (CRC Press, 2009).

[4] Brillo J, Egry I, Ho I (2006). Density and thermal expansion of liquid Ag-Cu and Ag-Au alloys. Int. J. Thermophys..

[5] Paradis P-F, Ishikawa T, Koike N (2008). Density of liquid gold measured by a non-contact technique. Gold. Bull..

[6] Egry I, Lohoefer G, Jacobs G (1995). Surface tension of liquid metals: results from measurements on ground and in space. Phys. Rev. Lett..

[7] Ofte D (1967). The viscosities of liquid Uranium, gold and lead. J. Nucl. Mater..

[8] Watanabe M, Adachi M, Uchikoshi M, Fukuyama H (2020). Densities of Pt-X (X: Fe, Co, Ni and Cu) binary melts and thermodynamics correlations. Fluid Ph. Equilibr..

[9] Jeon S (2022). Precise density measurement and its uncertainty evaluation for refractory liquid metals over 3000 K using electrostatic levitation. Metrologia.

[10] Morohoshi K, Uchikoshi M, Isshiki M, Fukuyama H (2011). Surface tension of liquid Iron as functions of oxygen activity and temperature. ISIJ Int..

[11] Nawer J, Matson DM (2023). Quantifying facility performance during thermophysical property measurement of liquid Zr using Electrostatic Levitation. High. Temp.- High. Press..

[12] Sachtler WMH, Dorgelo GJH, Holscher AA (1966). The work function of gold. Surf. Sci..

[13] Baker E, Nawer J, Xiao X, Matson DM (2020). MHD surrogate model for convection in electromagnetically levitated molten metal droplets processed using the ISS-EML facility. NPJ Microgravity.

[14] Bracker GP (2020). The effect of flow regime on surface oscillations during electromagnetic levitation experiments. High. Temp. High. Press.

[15] Brillo J, Egry I, Giffard HS, Patti A (2004). Density and thermal expansion of liquid Au–Cu alloys. Int. J. Thermophys..

[16] Zhang B, Li X, Li D (2013). Assessment of thermal expansion coefficient for pure metals. CALPHAD: Comput. Coupl. Ph. Diagr. Thermochem..

[17] Ishikawa T, Koyama C, Saruwatari H, Paradis P-F (2022). Status of the electrostatic levitation furnace in the ISS surface tension and viscosity measurements. Int. J. Microgravity Sci. Appl..

[18] Douady S (1990). Experimental study of the Faraday Instability. J. Fluid Mech..

[19] Lohöfer G (2005). Electrical resistivity measurement of liquid metals. Meas. Sci. Technol..

[20] Gebhart E, Becker M (1951). Über die innere Reibung flüssiger Gold-Silber-Legierungen. J. Mater. Res.

[21] Tempus, Team. Containerless processing in space: Recent results. *Materials and Fluids under Low Gravity: Proceedings of the IX*^*th*^*European Symposium on Gravity-Dependent Phenomena in Physical Sciences*, *held in Berlin, Germany,* Vol. 464 (eds. Ratke, L., Walter, H. & Feuerbacher, B.) 233–252 (Springer, New York, 1996).

[22] Xiao X, Brillo J, Lee J, Hyers RW, Matson DM (2021). Impact of convection on the damping of an oscillating droplet during viscosity measurement using the ISS-EML facility. NPJ Microgravity.

[23] JCGM/WG1. *Evaluation of measurement data—Guide to the expression of uncertainty in measurement*. (Joint Committee for Guides in Metrology, 2008).

[24] Okada JT, Ishikawa T, Watanabe Y, Paradis P-F (2010). Surface tension and viscosity of molten vanadium measured with an electrostatic levitation furnace. J. Chem. Thermodyn..

[25] Haumesser PH, Bancillon J, Daniel M, Perez M, Garandet J-P (2002). High-temperature contactless viscosity measurements by the gas–film levitation technique: application to oxide and metallic glasses. Rev. Sci. Instrum..

[26] Xiao X, Hyers RW, Wunderlich RK, Fecht H-J, Matson DM (2018). Deformation induced frequency shifts of oscillating droplets during molten metal surface tension measurement. Appl. Phys. Lett..

[27] Egry I, Brillo J, Holland-Moritz D, Plevachuk Y (2008). The surface tension of liquid aluminium-based alloys. Mater. Sci. Eng. A..

[28] Tamaru H (2018). Status of the electrostatic levitation furnace (ELF) in the ISS-KIBO. Microgravity Sci. Technol..

[29] Pletser V (2015). European parabolic flight campaigns with Airbus ZERO-G: Looking back at the A300 and looking forward to the A310. Adv. Space Res.

[30] Lohöfer G (2018). High-resolution inductive measurement of electrical resistivity and density of electromagnetically levitated liquid metal droplets. Rev. Sci. Instrum..

